# The Asylums Board and Sick Children: Inhumanity or Progress?

**Published:** 1913-09-06

**Authors:** H. B. Morgan

**Affiliations:** Senior House Physician. West Ham and Eastern General Hospital


### THE EDITOR'S LETTER-BOX.

### The Asylums Board and Sick Children: Inhumanity or Progress?

*To the Editor of* The Hospital.

Sir,—Will you allow me to enter a mild protest against the tenor of the paragraph in this week's Hospital relating to the recent circular of the M.A.B. to Boards of Guardians? Objection was apparently only taken to the direct admission of cases to the excellently equipped Board's hospitals for Poor-Law children on the puerile ground of distance.

Public bodies are very slow to move, and it seems a pity that the influence of The Hospital should be thrown against such a step in the right direction, and still more that the scheme should be thoughtlessly stigmatised as inhuman.

The whole subject is a controversial one. It is best that these questions should be discussed openly and fairly and without frivolous recriminations in your columns than that progressive ideas for improvement in the Poor Law should be buried in committee discussion.

Perhaps The Hospital will open a controversy on the subject.—Yours faithfully,

H. B. Morgan, Senior House Physician.

West Ham and Eastern General Hospital,
August 30.

[Probably the best way to convince Dr. Morgan that the criticism to which he refers was not "thoughtless" will be the publication of further letters on the subject. Not wishing to deprive him of the controversy which he invites, we refrain from comment at present.—Ed. The Hospital.]

